# Pregnancy With Uterine Fibroids: Obstetric Outcome at a Tertiary Care Hospital of Central India

**DOI:** 10.7759/cureus.35513

**Published:** 2023-02-27

**Authors:** Amruta Choudhary, Saunitra A Inamdar, Urvashi Sharma

**Affiliations:** 1 Department of Obstetrics and Gynaecology, Datta Meghe Medical College, Datta Meghe Institute of Medical Sciences (Deemed to be University), Nagpur, IND; 2 Department of Obstetrics and Gynaecology, Jawaharlal Nehru Medical College, Datta Meghe Medical Institute of Science (Deemed to be University), Wardha, IND

**Keywords:** neonatal outcome, postpartum hemorrhage, maternal complications, pregnancy complications, uterine fibroids

## Abstract

Background: Uterine fibroids are the most frequent benign tumor of the female reproductive system, with a significantly lower frequency in pregnancy. This could be due to the fact that uterine fibroids are linked to infertility and low implantation rates following in vitro fertilization (IVF). The goal of this study was to look at the obstetrics outcomes of uterine fibroids and their consequences in a tertiary hospital.

Materials and methods: The current study was a observational cohort study that evaluated the cases of pregnancy with fibroid. Study was undertaken at the Department of Obstetrics and Gynecology (OBGY) at a medical college in central India and it was conducted over a period of nine months from 1st November 2021 to 31st July 2022. All pregnant women who had an ultrasonography (USG)-documented uterine fibroid diagnosed prenatally or antenatally were enrolled. All demographic information, laboratory and USG results were noted and their mode of delivery, obstetric complications, if any, and neonatal outcomes were evaluated.

Results: A total of 110 cases were enrolled as per inclusion and exclusion criteria. The majority of patients (42.73%) were in the 26- to 30-year-old age group. In this study, the majority of cases were carried to term (80.9%). The most prevalent mode of delivery was caesarean section (61.82%). Major complications during pregnancy were threatened preterm labor (21.82%), and blood transfusion (20.00%), whereas postpartum hemorrhage (PPH) occurred in 9.09% cases, and 47 patients (42.72%) were asymptomatic throughout pregnancy. Major neonatal outcomes in our study were neonatal intensive care unit (NICU) hospitalization (20%), required neonatal resuscitation (14.55%), and neonatal mortality occurring in 1.82% cases. Gestational age at termination of pregnancy, when compared with different characteristics of fibroid, like type (p value 0.663), location (p value 0.552) and number of fibroid (p value 0.112), did not show any significant association. Similarly, maternal complications also did not show significant association (p value >0.05) with different characters of fibroid.

Conclusion: Pregnancies with fibroid are high-risk pregnancies that are linked to difficulties throughout the antepartum, intrapartum, and postpartum periods, as well as increased chances of cesarean delivery and PPH.

## Introduction

Uterine leiomyoma is one of the most frequent benign tumors of the female reproductive system. It develops from the smooth muscle of the uterus. It affects 20-40% of women, although the estimated incidence during pregnancy is 0.1-3.9% [1,2]. The fact that uterine fibroid is linked to infertility and low implantation rates following in vitro fertilization (IVF) could explain the significantly lower prevalence in pregnancy [3]. The physical examination can only diagnose 42% of large fibroids (>5 cm) and 12.5% of smaller fibroids (3-5 cm) [3,4]. Ultrasound's ability to detect fibroids in pregnancy is much lower (1.4-2.7%), owing to the difficulties in distinguishing fibroids from normal myometrial thickness [5]. Pregnancy with fibroid is associated with complications like antepartum hemorrhage (APH), acute abdomen, red degeneration of fibroid, laparotomy, preterm labor, malpresentation, and malposition of fetus, postpartum hemorrhage (PPH), retention of the placenta, dysfunctional labor and, intrauterine growth restriction (IUGR) [5-7]. The majority of cases of complications of uterine fibroid with pregnancy require conservative management, whereas, some cases like pedunculated fibroid with torsion require emergency surgical intervention [2,5-7]. This study was carried out to evaluate the obstetrics outcomes of patients with uterine fibroids and their consequences in a tertiary hospital.

## Materials and methods

The present study was an observational cohort study undertaken at the Department of Obstetrics and Gynecology (OBGY) at a medical college in central India. The study was conducted over a period of nine months from 1st November 2021 to 31st July 2022. Institutional Ethics Committee, Shalinitai Meghe Hospital and Research Centre and Datta Meghe Medical College, Nagpur, issued approval DMMC(DU)/IEC/2021/16. In this study, we enrolled pregnant women who had an ultrasonography (USG)-documented uterine fibroid diagnosed prenatally or antenatally. All the data, including demography, antenatal and postdelivery details were collected retrospectively from their case files, without breaching the confidentiality of the patient. Pregnant women who had a previous cesarean section, surgery, uterine deformity, or chronic conditions such as hypertension and diabetes were excluded. The case record proforma included demographic information, antenatal/intrapartum/postpartum history (maternal age, parity, gravida, gestational age at enrollment and at delivery, number and size of fibroids), clinical examination findings, laboratory investigations, USG findings (fetal parameters, liquor, placental location and change in fibroid size or any complication), and the outcome. Obstetric issues if any, like preterm birth, premature rupture of membranes (PROM), malpresentation, placenta previa, placental abruption, low birth weight), mode of delivery, morbidity, and mortality related to the management of pregnancy with fibroids were documented. Neonatal outcomes like birth weight, APGAR score, neonatal resuscitation, and neonatal intensive care unit (NICU) admission were documented. Descriptive statistics were used in the statistical analysis. For discrete variables, data was imported into Microsoft Excel (Redmond, WA, USA) and presented as numbers and percentages and mean and standard deviation were given for quantitative data and multiple logistics regression analysis was done to show any association of perinatal outcome with characteristics of fibroid.

## Results

One hundred ten pregnant women who had USG findings of fibroid were enrolled in this study. The mean age of patients in study was 31.0 years with standard deviation of 4.93, but majority of patients (42.73%) were in the 26-30 years age group, and then in the 31-35 years age group (30.00%). 6.36% of patients were less than 25 years and 2.63% of patients were more than 40 years of age (Table 1).

**Table 1:** Age in years

Demographic characters	Number of cases	Percentage
Age (in years)		
19 – 25	7	6.36%
26 - 30	47	42.73%
31 – 35	33	30.00%
36 – 40	19	17.27%
≥ 41	4	3.64%

40.91% of patients were primigravida and 38.18% of patients were of second or third gravida and 20.91% of patients were of fourth gravida (Table 2).

**Table 2:** Gravida status

Gravida status	Number of cases	Percentage
Primigravida	45	40.91%
Gravida 2-3	42	38.18%
Gravida ≥ 4	23	20.91%

The majority of the patients had subserous fibroids (71.82%), and 20.91% of patients had submucous fibroids. The most common location of fibroid was the fundus region (77.27%) and 12.73% of patients had pedunculated fibroid. 50.91% of patients had two to three fibroids (Table 3).

**Table 3:** Features of uterine fibroid

Features of uterine fibroids	Number of cases	Percentage
Type of fibroid		
Intramural	8	7,27%
Submucous	23	20.91%
Subserous	79	71.82%
Location of fibroid		
Cervix	5	4.55%
Fundus	85	77.27%
Tubes (cornual)	6	5.45%
Pedunculated	14	12.72%
Number of fibroids		
1	30	27.27%
2–3	56	50.91%
>=4	24	21.82%

In this study, the majority of cases were delivered at term (80.9%). The most common mode of delivery was cesarean section (61.82%), followed by vaginal delivery (32.71%), including instrumental and assisted breech delivery. The major indication for cesarean section was for PROM with a low Bishop score (33.82%) and malpresentation (17.65%) (Table 4). Gestational age at termination of pregnancy when compared with type of fibroid (p value 0.663), location of fibroid (p value 0.552), and number of fibroid (p value 0.112) did not show any significant relationship.

**Table 4:** Obstetric outcome LSCS: lower segment cesarean section, PROM: premature rupture of membranes

Pregnancy outcome	Number of cases	Percentage
Gestational age at termination of pregnancy		
≤ 20 weeks	3	2.73%
21-32 weeks	4	3.64%
33-37 weeks	14	12.73%
37-40 weeks	83	75.45%
≥ 40 weeks	6	5.45%
Delivery mode		
Cesarean section	68	61.82%
vaginal normal delivery	29	26.36%
Instrumental delivery	4	3.64%
Assisted Breech delivery	2	1.82%
Hysterotomy	4	3.64%
Suction and evacuation	3	2.73%
Indication for LSCS (n=68)		
PROM with poor Bishop score	23	33.82%
Placenta previa	6	8.82%
Uterine inertia	10	14.71%
Fetal distress	10	14.71%
Non progressive labor	7	10.29%
Malpresentation	12	17.65%

Major complications during pregnancy were threatened preterm labor (21.82%) and threatened abortion (16.36%), whereas PPH occurred in 9.09% of cases, and blood transfusion was required in 20% of cases. Forty-seven patients (42.72%) were asymptomatic throughout pregnancy (Table 5). When maternal complications like antepartum bleeding, PPH, or threatened preterm labour, when analysed as per type of fibroid, location or number of fibroids, did not show any significant association (i.e. p value <0.05) by multiple regression analysis.

**Table 5:** Complication during pregnancy

Complications	Number of cases	Percentage
Threatened preterm labour	24	21.82%
Blood transfusion	22	20%
Postpartum hemorrhage	10	9.09%
Antepartum bleeding	12	10.91%
Threatened miscarriage	18	16.36%
Admission for pain in abdomen	6	5.45%
Laparotomy for abdominal pain	0	0

Neonatal outcomes in our study were low birth weight (16.36%), low APGAR score at five minutes (10.90%), required neonatal resuscitation (14.54%) and required NICU hospitalization (20%), and neonatal mortality occurred in 1.81% of cases (Table 6).

**Table 6:** Fetal Outcome NICU: neonatal intensive care unit

Fetal Outcome	Number of cases	Percentage
Abortion	6	5.45%
Low birth weight	18	16.36%
Low APGAR Score at 5 minutes	12	10.91%
Required neonatal resuscitation	16	14.55%
Required NICU admission	22	20%
Neonatal death	2	1.82%

## Discussion

The global incidence of pregnancy with fibroid uterus is on a rising trend because of delays in conception and rising maternal ages. The size and location of the leiomyoma are the two most important parameters that predict morbidity in pregnancy. If the placenta is implanted directly over the fibroid or adjacent to it, complications occurred are abortion, IUGR, APH, and PPH [8,9]. A tumor in the cervix or lower uterine segment, on the other hand, may hinder labor. Malpresentations, particularly breech presentations, are common, and the size and placement of the leiomyoma might help forecast the risk [10]. Indiscriminate use of electrosurgical energy and avoiding the multilayer closure of the myoma bed led to increased incidences of uterine rupture [11,12].

The impact of USG-diagnosed multiple or big (>=5 cm) fibroids on obstetric outcomes was investigated by Ciavattini et al. [5]. The study included 219 women who had uterine fibroids. Women with numerous fibroids (n=34) had a significantly greater rate of preterm delivery, cesarean section, and breech presentation when compared to women without fibroids. Preterm birth and preterm premature rupture of membranes (PPROM) were more common in women with big fibroids (n=48). Multiple fibroids are linked to a higher risk of preterm birth and cesarean delivery, while large fibroids are linked to a higher risk of PPROM, according to their findings. In most cases, fibroids in pregnancy are treated conservatively. In our study, cesarean section occurred in 61.82% whereas PROM occurred in 33.82% and preterm delivery occurred in 12.73% of cases. Though we had increased cesarean section and preterm delivery in our study, we did not find any significant association between type of delivery and location and number of fibroid.

Pullemalla et al. [13] studied 50 patients and observed that pregnant women with fibroid were at a higher risk of complications antenatally, intranatally, and also in the postpartum period. Pregnancy outcomes were abortion in two cases, delivery by cesarean section in 44 cases and four had normal deliveries. Thirty percent of patients had threatened miscarriage, and one case had PPH and needed a blood transfusion. In our study, cesarean section occurred in 61.82% and PROM was the most common indication for cesarean section.

Dasgupta et al. [14] studied the complications in pregnancy with large uterine fibroid. There was malpresentation in 60% of cases, and 87% of patients required blood transfusion. In 13.33% of cases, required bilateral prophylactic internal iliac artery ligation followed by myomectomy during the delivery of the baby. In our study, malpresentation occurred in 17.65% of cases.

Zhao et al. [15] conducted a multicenter investigation. In a study of 112,403 women, 3,012 (2.68%) were found to have at least one fibroid. Furthermore, the fibroid's location either intramural, submucosal or subserosal has a statistically significant impact on the probability of PPH (5.6% subserosal vs 4.7% submucosal vs 8.6% intramural). In our study, PPH occurred in 9.09% of cases, however, we have not found any significant relation (p value 0.086) between fibroid location and PPH.

In a study by Sundermann et al. [16], 4,622 pregnant women with a singleton pregnancy were evaluated and 475 of them had at least a single fibroid (10.3%). 352 pregnant women resulted in preterm birth (7.6%). On comparing the incidence of preterm and term birth, the prevalence of fibroid was similar in both groups (10.2% vs.10.3%). After considering the confounding factors, it was observed that preterm birth was not associated with uterine fibroid in pregnancy. Whereas, in our study, we had preterm delivery in 12.72% of cases. On analysis, gestational age at termination of pregnancy did not show any significant relation with type of fibroid (p value 0.063), location of fibroid (p value 0.552) or number of fibroid (p value 0.023)

In our study, though presence of uterine fibroid in pregnacy is associated with increased rate of cesarean section (61.82%) and required blood transfusion in 20% patients. Perinatal outcome did not show any significant association with type, location and number of fibroid on multiple regression analysis. This may be due to small sample size, which is one of the limitations of our study. Study was conducted only at one site and data were collected retrospectively from case files. High-risk pregnancy is definitely caused by presence of uterine fibroid along with pregnancy and majority of previous studies concluded the same. But further studies are required for establishing the association of type and size of fibroid with the perinatal outcome. Other limitations of the study included were all demographic characteristics of patients (BMI status, addictions like smoking) were not compared with the characteristics of fibroid.

## Conclusions

In the modern era of medicine, many people had their ultrasonography report done for some other reason. Thus, there is an increased diagnosis of uterine fibroid. Pregnancy with uterine fibroid are many times asymptomatic and is an incidental finding. Fibroid in pregnancy can be associated with abortion in early gestation and PROM in term gestation and PPH in the postpartum period. There is also an increased risk of malpresentation. The rate of cesarean section is high in pregnant patients with uterine fibroid. Thus, uterine fibroids in pregnancy make a normal pregnancy into a high-risk one. However, we did not find any significant association between type, location and number of fibroid with particular maternal complications. Thus, uterine fibroid in pregnancy should be followed up very frequently and need special attention in form of close monitoring of patients as well as the fetus.
